# The usefulness of noninvasive liver stiffness assessment using shear-wave elastography for predicting liver fibrosis in children

**DOI:** 10.1186/s12880-021-00601-8

**Published:** 2021-04-12

**Authors:** Seunghyun Lee, Young Hun Choi, Yeon Jin Cho, Seul Bi Lee, Jung-Eun Cheon, Woo Sun Kim, Jae Sung Ko, Jaemoon Koh, Gyeong Hoon Kang

**Affiliations:** 1 Department of Radiology, Seoul National University Hospital, 101 Daehak-ro, Jongno-gu, Seoul, 03080 Republic of Korea; 2 Department of Radiology, Seoul National University College of Medicine, 103 Daehak-ro, Jongno-gu, Seoul, 03080 Republic of Korea; 3 Institute of Radiation Medicine, Seoul National University Medical Research Center, 103 Daehak-ro, Jongno-gu, Seoul, 03080 Republic of Korea; 4 Department of Pediatrics, Seoul National University Hospital, 101 Daehak-ro, Jongno-gu, Seoul, 03080 Republic of Korea; 5 Department of Pathology, Seoul National University Hospital, 101 Daehak-ro, Jongno-gu, Seoul, 03080 Republic of Korea

**Keywords:** Child, Liver diseases, Elasticity imaging techniques, Fibrosis

## Abstract

**Background:**

Pediatric patients with liver disease require noninvasive monitoring to evaluate the risk of fibrosis progression. This study aimed to identify the significant factors affecting liver stiffness values using two-dimensional shear-wave elastography (2D-SWE), and determine whether liver stiffness can predict the fibrosis stage of various childhood liver diseases.

**Methods:**

This study included 30 children (22 boys and 8 girls; mean age, 5.1 ± 6.1 years; range, 7 days–17.9 years) who had undergone biochemical evaluation, 2D-SWE examination, histopathologic analysis of fibrosis grade (F0 to F3), assessment of necroinflammatory activity, and steatosis grading between August 2016 and March 2020. The liver stiffness from 2D-SWE was compared between fibrosis stages using Kruskal–Wallis analysis. Factors that significantly affected liver stiffness were evaluated using univariate and multivariate linear regression analyses. The diagnostic performance was determined from the area under the receiver operating curve (AUC) values of 2D-SWE liver stiffness.

**Results:**

Liver stiffness at the F0-1, F2, and F3 stages were 7.9, 13.2, and 21.7 kPa, respectively (*P* < 0.001). Both fibrosis stage and necroinflammatory grade were significantly associated with liver stiffness (*P* < 0.001 and *P* = 0.021, respectively). However, in patients with alanine aminotransferase (ALT) levels below 200 IU/L, the only factor affecting liver stiffness was fibrosis stage (*P* = 0.030). The liver stiffness value could distinguish significant fibrosis (≥ F2) with an AUC of 0.950 (cutoff value, 11.3 kPa) and severe fibrosis (F3 stage) with an AUC of 0.924 (cutoff value, 18.1 kPa). The 2D-SWE values for differentiating significant fibrosis were 10.5 kPa (≥ F2) and 18.1 kPa (F3) in patients with ALT levels below 200 IU/L.

**Conclusion:**

The liver stiffness values on 2D-SWE can be affected by both fibrosis and necroinflammatory grade and can provide excellent diagnostic performance in evaluating the fibrosis stage in various pediatric liver diseases. However, clinicians should be mindful of potential confounders, such as necroinflammatory activity or transaminase level, when performing 2D-SWE measurements for liver fibrosis staging.

**Supplementary Information:**

The online version contains supplementary material available at 10.1186/s12880-021-00601-8.

## Background

Pediatric liver diseases have a wide range of etiologies, including congenital, metabolic, toxic, and infectious diseases, as well as a fatty liver [1]. Prolonged, repeated hepatocellular injury can lead to liver fibrosis, especially in pediatric patients who may exhibit an unpredictable progression [2]. Therefore, pediatric patients with liver disease require monitoring for the likelihood of liver fibrosis progression similar to adult patients.

Both liver function biochemical assessment and ultrasound (US) examination are used for liver fibrosis monitoring, but liver biopsy, which is performed only if necessary, is considered the gold standard despite its invasiveness and potential for sampling errors [1]. Various noninvasive monitoring methods, such as serum biochemical markers and quantitative liver elastography assessments, are promising alternatives to liver biopsy. Previous studies have shown that various serum biochemical indicators, such as aspartate aminotransferase (AST), alanine aminotransferase (ALT), AST to platelet ratio index (APRI), AST and ALT ratio (AAR), and fibrosis index based on the 4 factor (FIB-4) score, could be candidate markers in adult chronic liver patients [3–5]. However, further evaluation of their clinical utility for liver fibrosis is needed in pediatric patients because of their different etiologies [6, 7].

Noninvasive US elastography, which measures liver stiffness mainly on the basis of fibrosis, could be another option for monitoring fibrosis, in pediatric liver diseases [8]. The two-dimensional shear-wave elastography (2D-SWE) value (obtained in m/s, and then converted in Young’s module unit in kilopascals [kPa] by making some assumptions) offers advantages in quantitative assessment, and several studies have reported the clinical utility of 2D-SWE to assess liver fibrosis in chronic liver disease [9–12]. However, only a few studies have used 2D-SWE to evaluate the clinical significance of 2D-SWE liver stiffness in pediatric patients with liver diseases, even though many studies have evaluated the advantages of SWE in adult populations with non-alcoholic fatty liver disease (NAFLD), hepatitis, autoimmune hepatitis, or other liver diseases [12–16].

Therefore, this study evaluated the significant factors influencing liver stiffness values in 2D-SWE and determined whether liver stiffness can predict the fibrosis stage of various childhood liver diseases.

## Methods

Following the Declaration of Helsinki, the study was approved as a retrospective human study by the Institutional Review Board of Seoul National University Hospital (No. 2005-211-1127). Informed consent from the patients, parents, or guardians was waived accordingly.

### Patient population

Patients with suspected liver disease were referred for liver biopsies to assess their histopathological conditions between August 2016 and March 2020. We retrospectively reviewed the picture archiving and communication system (Infinitt; Infinitt Healthcare, Korea) database. The patients in our study were included as follows: (1) patients aged < 18 years; (2) patients who had undergone both 2D-SWE examination and serological biochemical marker evaluation before the liver biopsy; and (3) patients who had obtained histopathological results through a liver biopsy for various liver diseases.

**Fig. 1:**
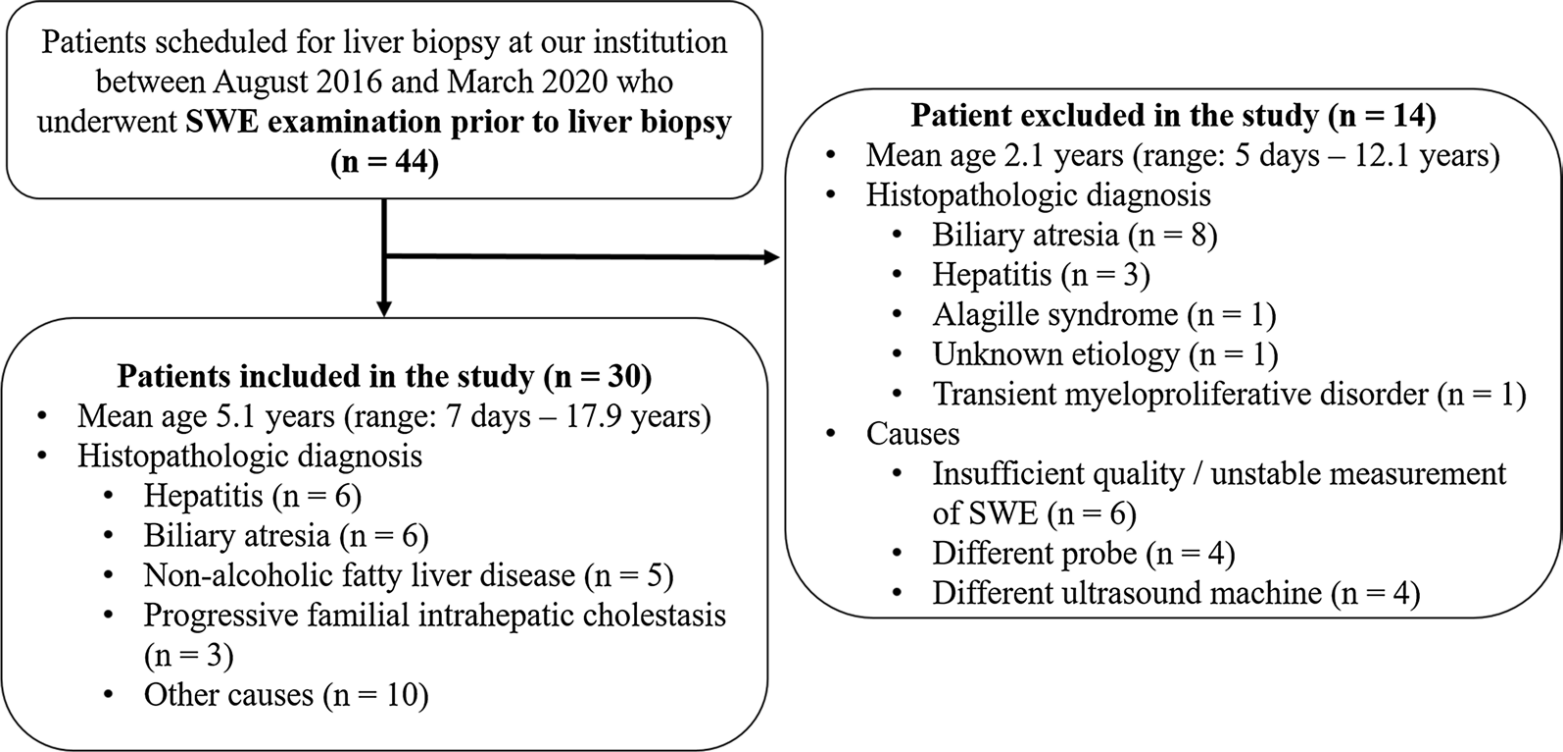
Flowchart illustrating the selection of the study population. We retrospectively reviewed the picture archiving and communication system database for the period between August 2016 and March 2020. Of the 44 potential candidates who underwent two-dimensional shear-wave elastography (SWE) prior to liver biopsy, 14 were excluded for the following reasons: insufficient quality of SWE (n = 6), use of different probes (n = 4), and use of different US machines (n = 4)

Fourteen patients were excluded from this study for the following reasons: insufficient quality of SWE as explained in the 2D-SWE Liver Stiffness Examination section (n = 6), use of different probes such as the high-frequency linear probes (n = 4), and the cases of different US machines due to the retrospective study design (n = 4). The number of excluded patients was 14, with a mean age of 2.1 ± 4.1 years (range 5 days–12.1 years). The most common histopathologic diagnoses in the excluded patients were biliary atresia (BA) (n = 8), and the others included hepatitis (n = 3), Alagille syndrome (n = 1), undiagnosed disease (n = 1), and transient myeloproliferative disorder (n = 1) (Fig. 1).

Serum biochemical analysis was performed on all patients within 1 week before liver biopsy. Serum levels of total bilirubin, alkaline phosphatase, AST, ALT, γ-glutamyl transpeptidase, direct bilirubin, and albumin were measured, as were prothrombin time and platelet counts. We calculated the APRI, AAR, and FIB-4 scores [3, 4, 17].

### 2D-SWE liver stiffness examination

All included patients had available results of liver US examination, including 2D-SWE elastography, without any anesthesia at least 3 days before or on the day of liver biopsy. The 2D-SWE examinations were performed by two experienced pediatric radiologists who were randomly assigned (S.L. and Y.H.C. with 6 and 13 years of pediatric US examination experience, respectively) using the Aixplorer machine (SuperSonic Imagine SA, France) with a convex probe (SC6–1). The 2D-SWE examinations were performed according to the US elastography guideline previously reported.8 Patients had maintained a fasting state for a minimum of 2 h before US and 2D-SWE examinations and for a minimum of 4 h to evaluate gallbladder morphology if BA was suspected.

The patient was placed in a supine position to visualize the liver's right lobe, and we set a 2.0 × 2.0-cm color-coded box at a distance of 1.0 cm away from the Glisson's capsule, avoiding large blood vessels. A 10 mm circular region of interest (ROI) was carefully placed over an evenly color-coded area in the SWE box. We obtained the liver stiffness values, standard deviations, and stability index (SI) values in the ROIs. All measurements were performed using SI, and only ROIs with SI values of 90% or higher were considered for evaluation [18, 19]. Information with SI calculation allowed the user to rule out low-quality signals before calculating the interquartile range (IQR). In this study, liver stiffness measurements were considered successful when ROIs with SI values of 90% or higher were obtained across the entire series of SWE images. Otherwise, the measurement was considered to have failed and of insufficient image quality. In addition, when the normalized value divided by the IQR/median value from a total of 10 measurements showed a variation of 30% or more, the liver stiffness measurement was regarded as insufficient quality of the dataset because of the high variable measurability of the stiffness value [18, 20]. Finally, we selected the median liver stiffness values among sufficiently qualified images and stable measured data for further analysis.

### Histopathologic analysis

Within 3 days of the US examination including 2D-SWE, the patients underwent percutaneous liver parenchymal biopsy with an 18-gauge core biopsy device (TSK Ace-cut; Japan) while maintaining mild to moderate sedation using intravenous sedative drugs. We performed a biopsy at the right lobe of the previously measured location for each patient, without any significant adverse events. According to our protocols, biopsy samples were obtained three times with a specimen length of 11 mm or two times with a specimen length of 22 mm.

Two pediatric pathologists (J.K., with 6 years of experience in liver histopathologic evaluation and G.H.K., over 20 years of experience in pediatric gastrointestinal pathology), who were not aware of the US results for SWE values, reviewed the specimens by consensus. We retrospectively reviewed the pathologic reports and assessed the stages of liver fibrosis using the METAVIR staging system. We staged fibrosis on a five-point ordinal scale from 0 to 4 as follows: F0, absent and F4, cirrhosis [13]. We evaluated the liver necroinflammatory activity grade from 0 to 3: A0, no activity and A3, severe activity [21]. We also graded the similar steatosis grade from 0 to 3: S0, no steatosis and S3, above the two-third fatty accumulation in the hepatocytes [22].

### Statistical analyses

Descriptive demographic data were expressed as mean ± standard deviation. The median liver stiffness values were compared among the fibrosis stages using the Kruskal–Wallis analysis. The significant factors affecting liver stiffness were assessed using linear regression methods by univariate and multivariate analyses.

The areas under the receiver operating characteristic curves (AUCs) were analyzed to assess the diagnostic performance of 2D-SWE liver stiffness values for the presence of significant fibrosis (≥ F2) and advanced fibrosis (F3). We also calculated the optimal cutoff values at the highest Youden index and identified the sensitivity, specificity, positive predictive values, and negative predictive values.

Statistical analyses were performed using SPSS Statistics (version 21.0; IBM Corp., USA).

## Results

### Clinical characteristics

A total of 30 patients were included in our study, and the average patient’s age was 5.1 ± 6.1 years (range 7 days–17.9 years). There were 22 male patients (73.3%) in our study, and none of the patients had ascites.

Table 1 summarizes the etiology of liver disease and the results of the serological index. The histological diagnoses were as follows: hepatitis (n = 6), BA (n = 6), NAFLD (n = 5), and others (n = 13). The AST and ALT values showed a wide range in our study, which were 384.2 ± 577.6 and 440.3 ± 655.9 IU/L. The serologic indices such as APRI, AAR, and FIB-4 were 5.0 ± 8.4, 1.3 ± 1.2, and 0.4 ± 0.8, respectively.

**Table 1:** Clinical characteristics

Parameters	Patients (n = 30)
Age (years, mean ± SD) [range]	3.6 ± 5.5 [7 days to 17.9 years]
Sex (n, male:female)	22:8
Etiology of liver disease (%)	
Hepatitis	6 (20.0)
Biliary atresia	6 (20.0)
Non-alcoholic fatty liver disease	5 (16.7)
Others^†^	13 (43.3)
Serologic Index	
AST (mean ± SD) (IU/L) [range]	384.2 ± 577.6 [32–2946]
ALT (mean ± SD) (IU/L) [range]	440.3 ± 655.9 [12–3121]
APRI (AST to platelet ratio index) (mean ± SD) [range]	5.0 ± 8.4 [0.3–42.1]
AAR (AST to ALT ratio) (mean ± SD) [range]	1.3 ± 1.2 [0.2–5.0]
FIB-4 (fibrosis-4 score) (mean ± SD) [range]	0.4 ± 0.8 [0.0–3.9]
Grade of fibrosis (%)	
F0-1 (none or mild)	13 (43.3)
F2-3 (moderate or severe)	17 (56.7)
Necroinflammatory activity (%)	
A0-1 (none or minimal)	18 (60.0)
A2-3 (mild or moderate)	12 (40.0)
Degree of steatosis (%)	
S0-1 (none or mild < 33%)	26 (86.7)
S2-3 (moderate or severe, ≥ 33%)	4 (13.3)

^†^Progressive familial intrahepatic cholestasis (n = 3), glycogen storage disease (n = 2), hemosiderosis (n = 2), hemophagocytic lymphohistiocytosis (n = 2), autoimmune hepatitis (n = 2), congenital hepatic fibrosis (n = 1), and carnitine palmitoyltransferase I deficiency (n = 1). SD, standard deviation; AST, aspartate aminotransferase; IU, international units; ALT, alanine aminotransferase; APRI, AST to platelet ratio index; AST, aspartate aminotransferase; FIB-4, fibrosis-4 score

The fibrosis grades, necroinflammatory activity, and steatosis grades are summarized in Table 1. In all analyses, we considered the F0 and F1 stages to be identical. Only two patients showed no signs of fibrosis (F0) on histopathological analysis. One of these patients had hemosiderosis, and the other had a very rare metabolic liver disease, namely, carnitine palmitoyltransferase I deficiency. Therefore, we included these patients with F0 stage fibrosis in the F1 stage fibrosis group for statistical analysis, and the total number of patients in this combined F0-1 stage fibrosis group was 13. Subsequently, patients in fibrosis stages were divided into two subgroups: no or mild hepatic fibrosis (F0 and F1; n = 13) and significant hepatic fibrosis (F2 and F3; n = 17). Significant necroinflammatory activity (A2 and A3; n = 12) and steatosis grade (S2 and S3; n = 4) were also found as histopathologic abnormalities (Table 1).

A total of 16 patients with ALT levels below 200 were included in the subgroup analysis to minimize the confounding effect due to the high transaminase levels (Additional file 1: Table S1). The histological diagnoses were as follows: BA (n = 5), NAFLD (n = 4), hepatitis (n = 2), and others (n = 5). The AST and ALT values in the subgroup were 139.4 ± 137.1 and 97.6 ± 56.5 (IU/L). The serologic indices such as APRI, AAR, and FIB-4 were 1.8 ± 2.8, 1.9 ± 1.5, and 0.5 ± 1.0, respectively. The degree of fibrosis was equally distributed as follows: F0 and F1 (n = 8), and F2 and F3 (n = 8). Significant necroinflammatory activity (A2 and A3; n = 6) and steatosis grade (S2 and S3; n = 3) were also observed in the subgroup.

### Significant affecting factors to liver stiffness values

For 2D-SWE liver stiffness measurements, Table 2 summarizes the median liver stiffness values according to the fibrosis stage. There was a significant difference in liver stiffness values among the F0-1, F2, and F3 stages (*P* < 0.001). Table 3 shows the histologic, demographic, and serologic factors influencing 2D-SWE liver stiffness values. In the univariate analysis, fibrosis grade (odds ratio [OR] 4.064; 95% confidence interval [CI] 2.010–6.117; *P* < 0.001) and necroinflammatory grade (OR 2.189; 95% CI 0.099–4.280; *P* = 0.041) were associated with 2D-SWE liver stiffness values. In the multivariate linear regression analysis, fibrosis grade (OR 4.356, 95% CI 2.618–6.095; *P* < 0.001) and necroinflammatory grade (OR 2.207, 95% CI 0.365–4.050; *P* = 0.021) were significantly associated with liver stiffness.

**Table 2:** Median liver stiffness values in fibrosis stages

Variable	Fibrosis stages			*P* value*
	F0-1 (n = 13)	F2 (n = 11)	F3 (n = 6)	
Liver stiffness, kPa (IQR)	8.2 (7.3–10.9)	13.2 (12.6–15.8)	21.7 (17.4–23.7)	< 0.001

IQR is presented in parentheses. IQR, interquartile range. *P value was determined using Kruskal–Wallis analysis

**Table 3:** Factors affecting liver stiffness value determined by 2D-SWE

Characteristics	Univariate			Multivariate		
	Coefficient	95% CI	*P* value	Coefficient	95% CI	*P* value
Fibrosis Stage	4.064	2.010 to 6.117	< 0.001	4.356	2.618 to 6.095	< 0.001
Necroinflammatory activity	2.189	0.099 to 4.280	0.041	2.207	0.365 to 4.050	0.021
Steatosis grade	1.316	− 1.496 to 4.129	0.341	–		
Age (years)	-0.209	− 0.600 to 0.182	0.280	–		
Sex	-0.740	− 3.732 to 2.252	0.612	–		
APRI	-0.043	− 0.207 to 0.121	0.593	–		
AAR	0.235	− 0.990 to 1.460	0.694	–		
FIB-4	0.959	− 1.268 to 3.187	0.381	–		

2D-SWE, two-dimensional shear-wave elastography; CI, confidence interval; APRI, AST to platelet ratio index; AAR, AST to ALT ratio; FIB-4, fibrosis-4 score

In the subgroup analysis, the factors affecting liver stiffness values were fibrosis stage (OR 5.189; 95% CI 2.872–7.506; *P* < 0.001), necroinflammatory activity (OR 4.594; 95% CI 1.458–7.730; *P* = 0.007), and AAR (OR 2.078; 95% CI 0.472–3.683; *P* = 0.015) in the univariate linear regression analysis. Multivariate linear regression analysis showed that only fibrosis stage was significantly associated with the liver stiffness value (OR 3.149; 95% CI 0.359–5.940; *P* = 0.030) (Additional file 1: Table S2).

### Diagnostic performance of liver stiffness predicting fibrosis

The 2D-SWE liver stiffness values above 11.3 kPa showed 94.1% sensitivity and 84.6% specificity in distinguishing significant fibrosis (≥ F2) from no or mild fibrosis (AUC = 0.950; 95% CI 0.803–0.996; *P* < 0.001). At the advanced fibrosis stage F3, a liver stiffness value greater than 18.1 kPa showed 83.3% sensitivity and 100.0% specificity for differentiating fibrosis stages (AUC = 0.924; 95% CI 0.766–0.989; *P* < 0.001) (Table 4). In the subgroup analysis, the 2D-SWE values for differentiating significant fibrosis were 10.5 kPa (≥ F2) and 18.1 kPa (F3) in patients with ALT below 200 (Additional file 1: Table S3).

**Table 4:** Diagnostic performance of 2D-SWE for liver fibrosis

Stage	Cutoff	AUC (95% CI)	Sensitivity (%)	Specificity (%)	PPV (%)	NPV (%)	*P* value*
F ≥ 2	> 11.3	0.950 (0.803–0.996)	94.1	84.6	84.2	90.9	< 0.001
F ≥ 3	> 18.1	0.924 (0.766–0.989)	83.3	100.0	100.0	96.0	< 0.001

2D-SWE = two-dimensional shear-wave elastography. Diagnostic accuracy of each variable in association with fibrosis stage. The performance of the selected best cutoff values was indicated. AUC = the area under the receiver operating curve. PPV, positive predictive value; NPV, Negative predictive value; *Determined using receiver operating characteristic curve analysis

**Fig. 2:**
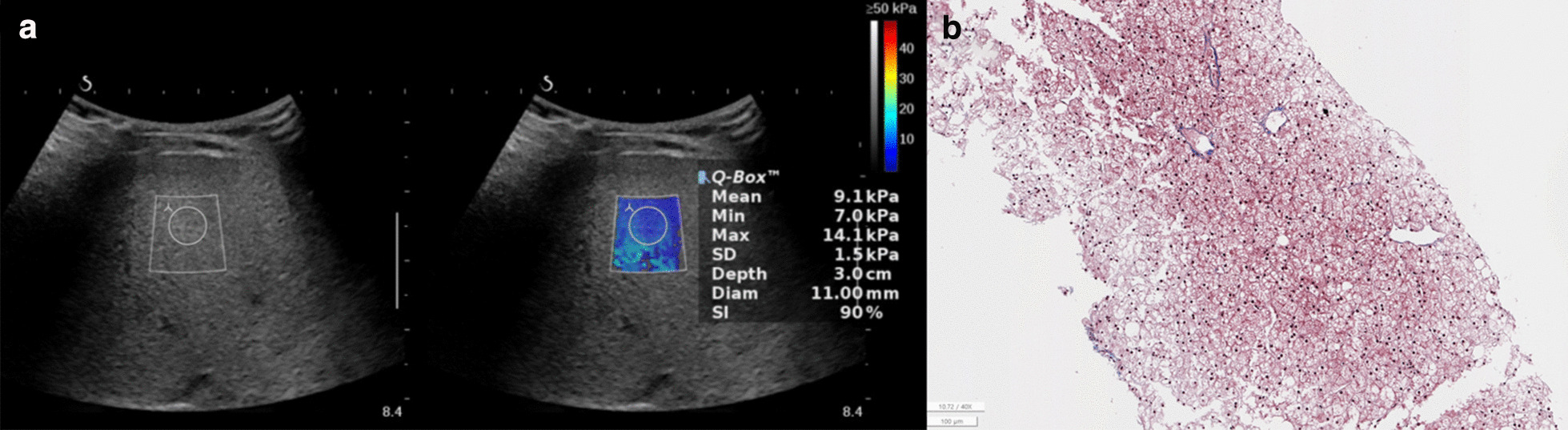
Glycogen storage disease of the liver in a 42-month-old boy. The serum biochemical marker levels were as follows: AST to platelet ratio index (APRI), 1.5; AST to alanine aminotransferase ratio (AAR), 0.9; and fibrosis-4 (FIB-4) score, 0.1. **a** Two-dimensional shear-wave elastography (2D-SWE) showing diffuse hyperechoic parenchyma with 9.1 kPa liver stiffness value. **b** Histopathologic specimen showing diffuse enlargement of hepatocytes with clear cytoplasm and enlargement of the fibrotic portal tract with METAVIR score, F1

**Fig. 3:**
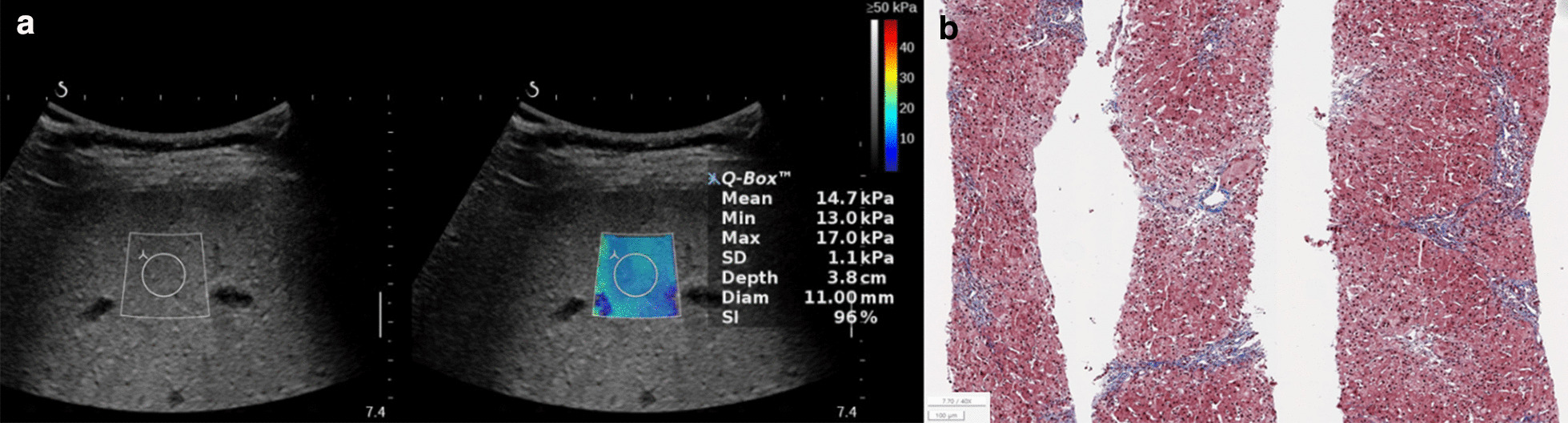
Hemophagocytic lymphohistiocytosis combined with the hepatitis in a 2.2-year-old boy. The serum biochemical marker levels were as follows: AST to platelet ratio index (APRI), 14.5; AST to alanine aminotransferase ratio (AAR), 0.8; and fibrosis-4 (FIB-4) score, 0.4. **a** Two-dimensional shear-wave elastography (2D-SWE) showing diffuse hyperechoic parenchyma with 14.7 kPa liver stiffness value. **b** Histopathologic specimen showing moderate lobular necroinflammatory activity and few portal fibrosis with METAVIR score, F2

**Fig. 4:**
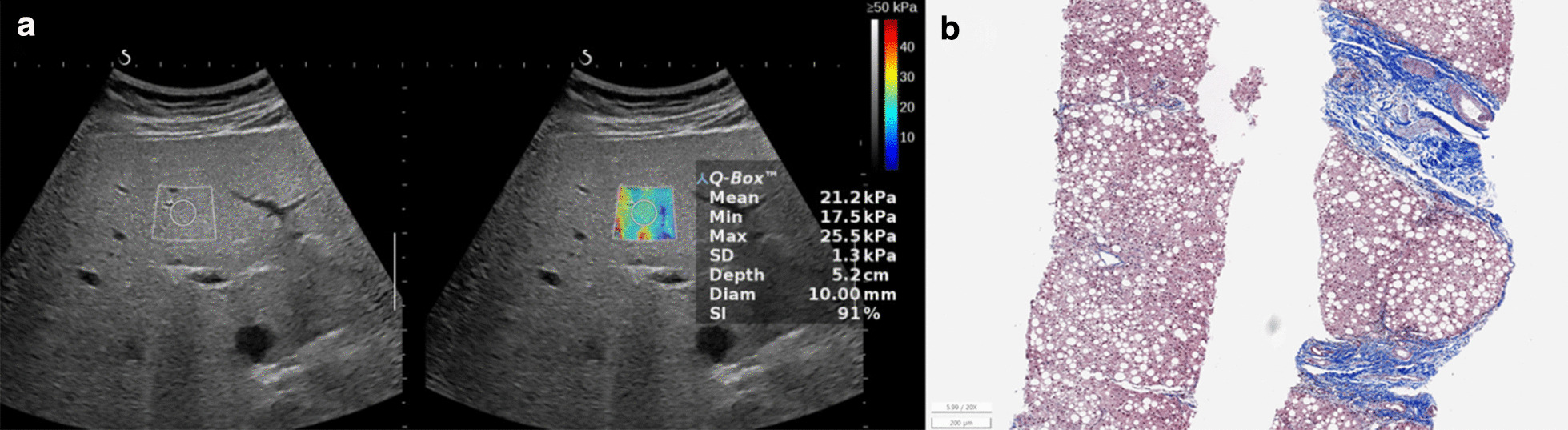
Non-alcoholic fatty liver disease in a 13.9-year-old boy. The serum biochemical marker levels were as follows: AST to platelet ratio index (APRI), 1.3; AST to alanine aminotransferase ratio (AAR), 0.4; and fibrosis-4 (FIB-4) score, 0.4. **a** Two-dimensional shear-wave elastography (2D-SWE) showing diffuse hyperechoic parenchyma with 21.2 kPa liver stiffness value. **b** Histopathologic specimen showing mild necroinflammatory activity, severe macrovesicular steatosis, and significant septal fibrosis with architectural distortion with METAVIR score, F3

Representative 2D-SWE and histopathologic specimens of the F1 case are shown in Fig. 2a, b. Each 2D-SWE and histopathologic sample of F2 and F3 are shown in Figs. 3a, b and 4a, b, respectively.

## Discussion

Our study demonstrated that liver stiffness measurements performed using 2D-SWE showed a significant association with liver fibrosis. Liver stiffness values may also be affected by necroinflammatory activity and transaminase levels. Therefore, a careful interpretation of liver stiffness is needed when fibrosis monitoring using 2D-SWE in pediatric patients with various liver diseases is carried out.

Liver fibrosis involves the progressive deposition of collagen material in the liver, which is a recovery response after chronic liver injury from various causes [23]. Noninvasive monitoring of mild to moderate liver fibrosis is important because cirrhosis, the end result of fibrosis, is irreversible. Previous studies have reported the performance of noninvasive diagnostic methods of liver fibrosis staging in adult patients [24–26]. A previous systematic review on 2D-SWE in liver fibrosis reported that the median cutoff value for presenting significant liver fibrosis (≥ F2) was 8.0 kPa (range 7.1–10.5) in adult patients [25]. Although liver elastography has been widely reported to have excellent diagnostic performance in adult patients, only a few studies have applied it in pediatric patients because of the difficulty in controlling and coordinating breathing in these patients despite the advantage offered by US evaluation. Recently, Kim et al. [15] had reported that liver stiffness value might be an excellent diagnostic value for evaluating liver fibrosis in children and adolescents in a meta-analysis of five studies, with a cutoff value of 9.4 kPa, a similar value as in adult patients.

Our study showed similar or slightly higher cutoff values for each fibrosis stage than a previous study on pediatric patients. However, our cutoff values should be compared to those of previously reported pediatric studies on a similar disease etiology [10]. Dhyani et al. reported that the cutoff value for the F2 stage would be 8.8 kPa in children and adolescents with diverse liver diseases [27]. Tutar et al. [28] and Franchi-Abella et al. [16] also reported that the cutoff values would be 10.4 kPa and 12.1 kPa in patients with NAFLD and early stage fibrosis, respectively. Therefore, in each study, the cutoff value may differ because of various causes and patient age groups. Our study could not demonstrate the cutoff value for any specific disease because of the diverse etiologies of pediatric liver diseases. Nevertheless, the advantage of this study was that it yielded meaningful results similar to existing data without sedation, even in neonates.

We also evaluated a variety of biochemical markers that could affect liver fibrosis. Previous studies on adult patients with viral hepatitis reported that the fibrosis stage and gamma-glutamyl transpeptidase levels could influence the liver stiffness value measured using 2D-SWE [24, 26]. Previous studies also reported that the APRI might be a diagnostic value in predicting the fibrosis stage in pediatric liver diseases [6, 29]. However, there was a controversy in the APRI diagnostic accuracy as a predictive indicator of liver fibrosis in children [6, 30]. In adults, these scoring systems could provide good diagnostic performance in not only NAFLD but also viral hepatitis [31]. Yang et al. [32] reported that the APRI and FIB-4 scores might be significant markers for predicting fibrosis in children with NAFLD. However, Mansoor et al. [6] reported that the APRI, AAR, and FIB-4 scores had poor diagnostic performance in identifying significant fibrosis in patients with NAFLD. Even recently developed fibrosis prediction systems, such as the improved liver fibrosis (ELF) scoring system, require unique serum markers, making their general use difficult [33].

Our study showed that serum biochemical markers such as the APRI, AAR, and FIB-4 scores provided a wide range because of the characteristics of various pediatric liver diseases. Therefore, these serologic markers did not affect liver stiffness values in the univariate linear regression analysis. However, when the ALT level was below 200 IU/L, AAR showed a significant association with the liver stiffness value despite the insignificance in the multivariate analysis. Therefore, a careful interpretation of liver stiffness is needed in various pediatric liver diseases because it is a confounding factor.

Among all the parameters explored in our study, fibrosis stage was the most relevant factor affecting 2D-SWE liver stiffness value. This could be because SWE can be applied to liver tissues to reflect the severity of liver fibrosis, even in various pediatric liver diseases. Another histopathologic factor, necroinflammatory grade, was relevant to liver stiffness values in pediatric liver diseases. All these histologic factors might be related to the stage of liver fibrosis. We have shown that both necroinflammatory and fibrotic stages based on histopathologic analysis can influence 2D-SWE liver stiffness values despite various liver diseases. However, only the fibrosis stage affected the liver stiffness values in the subgroup analysis. Therefore, the 2D-SWE liver stiffness value is a noninvasive marker suitable for clinical settings because of its correlation with the histologic fibrosis stage in various pediatric liver diseases.

Our study included patients with cholestatic liver disease in almost one-third of the study population. According to the EFSUMB guidelines, there was no evidence of the usefulness of 2D-SWE measurements in pediatric cholestatic liver disease patients due to the paucity of data.8 However, a recent expert opinion had suggested that the 2D-SWE value could be a valuable marker in cholestatic liver disease [34]. Another recent study reported that 2D-SWE value could be a valuable tool for predicting the stage of liver fibrosis in BA [35]. Nevertheless, large-scale cohort data for other cholestatic liver diseases have not yet been reported due to their infrequent incidence. As mentioned in previous reports, cholestatic conditions and tissue swelling or inflammation can lead to increased liver stiffness; therefore, careful interpretation is needed regarding the possibility of liver stiffness value overestimation [36]. Researchers using 2D-SWE measurements for liver fibrosis staging have to keep in mind the possible confounders such as necroinflammatory activity or transaminase level, but the various pediatric liver diseases could be evaluated through 2D-SWE measurements without regulating these confounders.

In terms of the influence of steatosis on liver stiffness measurements, there were no significant results on the effect of liver stiffness in our study. The 2D-SWE value for NAFLD in pediatric patients showed a higher liver stiffness value in a previous study. However, consistent results have not yet been reported for the effect of steatosis on 2D-SWE measurements obtained by US imaging in pediatric patients [37]. In fact, only four patients with significant steatosis were included in our study which was too small a sample size to assess the effect of liver stiffness. Therefore, a further large cohort study is needed to determine the effect of steatosis on liver stiffness.

Magnetic resonance elastography (MRE) might be an alternative diagnostic tool for the liver fibrosis stage. The diagnostic performance and reproducibility of MRE is generally higher than that of SWE examination [38–40]. However, compared to ultrasound-based elastography examination, MRE has a longer acquisition time, higher cost, and requires sedation, especially for uncooperative pediatric patients.

The present study had several limitations. We included only a small number of patients with a wide age range from neonates to adolescents who underwent 2D-SWE evaluation. A small number of patients in each fibrosis stage, with different etiologies and broad age ranges, could limit the results and clinical implications. However, to date, there is a paucity of published literature regarding the real-world clinical performance of SWE in the pediatric population. At present, SWE techniques employed in children are based mostly on the adult literature and expert opinion as opposed to scientific evidence; thus, continued research is still needed in this direction [34]. We believe that liver stiffness on SWE increases most often in response to increasing histologic fibrosis, although a variety of other pathologic and histologic changes may impact these measurements. However, there is a limitation in designing large-scale studies due to the small number of rare pediatric liver diseases. Efforts to accumulate data on these various diseases are required to evaluate the usefulness of the SWE technique. The clinical usefulness of 2D-SWE technology should be established for suspected patients with liver disease even in undiagnosed situations. Therefore, if liver stiffness obtained from 2D-SWE data on these various causes can be collected and estimated, this technique could be applied not only to NAFLD or hepatitis but also to fibrosis monitoring in various diffuse liver diseases. The advantage of our study is that even these various liver diseases showed statistically significant higher diagnostic accuracy of SWE values along the fibrosis stages. Nevertheless, further research is warranted on the clinical application of 2D-SWE in influencing the prognosis of suspected patients with liver diseases. The liver stiffness value on 2D-SWE could be overestimated according to the diverse etiologies such as severe inflammation, obstructive cholestasis, or congestive liver condition, which leads to an overestimation of the fibrosis stage [34]. Therefore, the liver stiffness cutoff values obtained in the present study might overestimate the stage of liver fibrosis rather than other cohorts and need the careful application of liver stiffness cutoff values that could only be applied to this series of patients.

## Conclusion

In conclusion, the liver stiffness values from 2D-SWE can be affected by both fibrosis and necroinflammatory grades and can be an excellent diagnostic tool for fibrosis stage evaluation, even in various liver disease types. However, 2D-SWE measurements for liver fibrosis staging have to keep in mind the possible confounders such as necroinflammatory activity or transaminase level accordingly.

## Supplementary Information

**Additional file 1.** Subgroup Analysis in Patients with Alanine Aminotransferase (ALT) < 200 (IU/L).

## Data Availability

The datasets used or analyzed during the current study are available from the corresponding author on reasonable request.

## Abbreviations

AAR: The aspartate aminotransferase to alanine aminotransferase ratio
ALT: Alanine aminotransferase
APRI: The aspartate aminotransferase to platelet ratio index
AST: Aspartate aminotransferase
AUC: Areas under the ROC curve
BA: Biliary atresia
FIB-4: Fibrosis-4 score
NAFLD: Non-alcoholic fatty liver disease
ROC: Receiver operating characteristic
SWE: Shear-wave elastography
US: Ultrasound
