# Supplementary material for: The usefulness of noninvasive liver stiffness assessment using shear-wave elastography for predicting liver fibrosis in children
Source: BMC Med Imaging. 2021 Apr 12;21:68. doi: 10.1186/s12880-021-00601-8 (PMC8040233; doi:10.1186/s12880-021-00601-8)
Supplement: Supplementary file 1 — Additional file 1. Subgroup Analysis in Patients with Alanine Aminotransferase (ALT) < 200 (IU/L). [file 12880_2021_601_MOESM1_ESM.docx]

**Supplementary Table 1. Clinical Characteristics in Patients with Alanine Aminotransferase (ALT) < 200 (IU/L)**

| **Parameters** | **Patients (n = 16)** |
| --- | --- |
| Age (years, mean ± SD) [range] | 6.0 ± 6.9 [7 days to 17.9 years] |
| Sex (n, male:female) | 12:4 |
| Etiology of liver disease (%) |  |
| Hepatitis | 2 (12.5) |
| Biliary atresia | 5 (31.3) |
| Non-alcoholic fatty liver disease | 4 (25.0) |
| Others^†^ | 5 (31.3) |
| Serologic Index |  |
| AST (mean ± SD) (IU/L) [range] | 139.4 ± 137.1 [32–594] |
| ALT (mean ± SD) (IU/L) [range] | 97.6 ± 56.5 [12–182] |
| APRI (AST to platelet ratio index) (mean ± SD) [range] | 1.8 ± 2.8 [0.3–12.0] |
| AAR (AST to ALT ratio) (mean ± SD) [range] | 1.9 ± 1.5 [0.5–5.0] |
| FIB-4 (fibrosis-4 score) (mean ± SD) [range] | 0.5 ± 1.0 [0.0–3.9] |
| Grade of fibrosis (%) |  |
| F0-1 (none or mild) | 8 (50.0) |
| F2-3 (moderate or severe) | 8 (50.0) |
| Necroinflammatory activity (%) |  |
| A0-1 (none or minimal) | 10 (62.5) |
| A2-3 (mild or moderate) | 6 (37.5) |
| Degree of steatosis (%) |  |
| S0-1 (none or mild < 33%) | 13 (81.3) |
| S2-3 (moderate or severe, ≥ 33%) | 3 (18.8) |

^†^ Glycogen storage disease (n = 1), hemosiderosis (n = 1), hemophagocytic lymphohistiocytosis (n = 1), autoimmune hepatitis (n = 1), and congenital hepatic fibrosis (n = 1).

**Supplementary Table 2. Factors Affecting Liver Stiffness Value Determined by 2D-SWE in Patients with Alanine Aminotransferase (ALT) < 200 (IU/L)**

| **Characteristics** | **Univariate** | | |  | **Multivariate** | | |
| --- | --- | --- | --- | --- | --- | --- | --- |
|  | Coefficient | 95% CI | *P*-value |  | Coefficient | 95% CI | *P*-value |
| Fibrosis Stage | 5.189 | 2.872 to 7.506 | < 0.001 |  | 3.149 | 0.359 to 5.940 | 0.030 |
| Necroinflammatory activity | 4.594 | 1.458 to 7.730 | 0.007 |  | 2.368 | -0.281 to 5.016 | 0.075 |
| Steatosis grade | -2.564 | -6.549 to 1.421 | 0.189 |  | - |  |  |
| Age (years) | -0.247 | -0.656 to 0.161 | 0.215 |  | - |  |  |
| Sex | 1.075 | -5.584 to 7.734 | 0.734 |  | - |  |  |
| ARPI | 0.397 | -0.653 to 1.448 | 0.431 |  | - |  |  |
| AAR | 2.078 | 0.472 to 3.683 | 0.015 |  | 0.909 | -0.396 to 2.214 | 0.155 |
| FIB-4 | 0.731 | -2.357 to 3.819 | 0.620 |  | - |  |  |

Note: 2D-SWE, two-dimensional shear-wave elastography; CI, confidence interval; APRI, AST to platelet ratio index; AAR, AST to ALT ratio; FIB-4, fibrosis-4 score.

**Supplementary Table 3. Diagnostic Performance of 2D-SWE for Liver Fibrosis in Patients with Alanine Aminotransferase (ALT) < 200 (IU/L)**

| **Stage** | **Cutoff** | **AUC (95% CI)** | **Sensitivity (%)** | **Specificity (%)** | **PPV (%)** | **NPV (%)** | ***P*-value**^*^ |
| --- | --- | --- | --- | --- | --- | --- | --- |
| F ≥ 2 | >10.5 | 0.953 (0.720–1.000) | 100.0 | 87.5 | 88.9 | 100.0 | <0.001 |
| F ≥ 3 | >18.1 | 0.872 (0.613–0.983) | 66.7 | 100.0 | 100.0 | 92.9 | 0.006 |

Note: 2D-SWE = two-dimensional shear-wave elastography. Diagnostic accuracy of each variable in association with fibrosis stage. The performance of the selected best cutoff values was indicated. AUC = the area under the receiver operating curve. PPV = positive predictive value. NPV = Negative predictive value; ^*^Determined using receiver operating characteristic curve analysis.
